# Review of the Southeast Asian species of the *Aenictus javanus* and *Aenictus philippinensis* species groups (Hymenoptera, Formicidae, Aenictinae)

**DOI:** 10.3897/zookeys.193.2768

**Published:** 2012-05-14

**Authors:** Weeyawat Jaitrong, Seiki Yamane

**Affiliations:** 1 Thailand Natural History Museum, National Science Museum, Technopolis, Khlong 5, Khlong Luang, Pathum Thani, 12120 Thailand; 2 Graduate School of Science and Engineering, Kagoshima University, Kagoshima. 890-0065 Japan

**Keywords:** *Aenictus javanus* group, *Aenictus philippinensis* group, army ants, taxonomy, new species, Southeast Asia

## Abstract

The Southeast Asian species of the *Aenictus javanus* and *Aenictus philippinensis* groups are revised. Six species (four named and two new species) of the *Aenictus javanus* group occurring in this area are: *Aenictus doydeei* Jaitrong & Yamane, 2011, *Aenictus duengkaei* Jaitrong & Yamane, **sp. n.**, *Aenictus javanus* Emery, 1896, *Aenictus longinodus* Jaitrong & Yamane, **sp. n.**, *Aenictus nishimurai* Terayama & Kubota, 1993, and *Aenictus piercei* Wheeler & Chapman, 1930. Four species (three named and one new species) are recognized in the *Aenictus philippinensis* group: *Aenictus pangantihoni* Zettel & Sorger, 2010, *Aenictus philippinensis* Chapman, 1963, *Aenictus punctatus* Jaitrong & Yamane, **sp. n.**, and *Aenictus rabori* Chapman, 1963. *Aenictus piercei* is removed from the members of the *Aenictus piercei* group sensu Jaitrong and Yamane (2011) and transferred to the *Aenictus javanus* group. Lectotypes and paralectotypes are designated for *Aenictus piercei* and *Aenictus rabori*. Size variation occurs among individuals from single colonies of the *Aenictus javanus* group, while the workers in the *Aenictus philippinensis* group are clearly monomorphic.

## Introduction

The genus *Aenictus* Shuckard, 1840 (subfamily Aenictinae) is one of the larger ant genera of the world. Curently 177 species and subspecies are listed (Bolton 2012). Jaitrong and Yamane (2011) established 12 species groups in the genus from the eastern part of the Oriental, Indo-Australian and Australasian regions, the groups being well defined on the basis of worker morphology.

The *Aenictus javanus* group is a small species group of the genus, defined by the following characteristics: antenna 10-segmented; mandible with 3 teeth including a large apical tooth; anterior margin of clypeus with several denticles; mesosoma in profile with dorsal margin almost flat. So far three species of the group, *Aenictus doydeei* Jaitrong & Yamane, 2011, *Aenictus javanus* Emery, 1896 and *Aenictus nishimurai* Terayama & Kubota, 1993 have been known, and all these are distributed only in Southeast Asia (Emery 1896, Forel 1909, Terayama and Kubota 1993, Jaitrong et al. 2011, Jaitrong and Yamane 2011). Jaitrong and Yamane (2011) included *Aenictus piercei* Wheeler & Chapman, 1930 in the “*Aenictus piercei* group”. However, after a careful examination of a specimen of *Aenictus piercei* kept together with the two syntypes, we decided to remove this species from this group that will be renamed in a different paper as the *Aenictus minutulus* group, and to transfer it to the *Aenictus javanus* group because in most respects this specimen and the syntypes possess a set of characteristics observed in the *Aenictus javanus* group.

The *Aenictus philippinensis* group is also a small species group of the genus, known only from the Philippines, consisting of three species: *Aenictus pangantihoni* Zettel & Sorger, 2010, *Aenictus philippinensis* Chapman, 1963 and *Aenictus rabori* Chapman, 1963 (Chapman 1963, Zettel and Sorger 2010, Jaitrong and Yamane 2011).

During our survey on the Asian *Aenictus* we found two new species of the *Aenictus javanus* group from Thailand and a new species of the *Aenictus philippinensis* group from Borneo and Java. In the present paper we revise these two groups in Southeast Asia and describe the three new species based on the worker caste. Morphological and bionomic information is presented for each species.

## Materials and methods

This study is mainly based on the materials deposited in the SKY Collection at Kagoshima University (Japan) and The Natural History Museum of the National Science Museum (Thailand). Syntypes or paratypes were examined for the five named species of the *Aenictus javanus* and *Aenictus philippinensis* groups. The holotype of *Aenictus doydeei* Jaitrong & Yamane, 2011 was also examined. The type material of *Aenictus philippinensis* Chapman, 1963 was not examined, but specimens from the type locality (Philippines, Negros) were examined.

Most morphological observations were made with a Nikon SMZ1000 stereoscope. Multi-focused montage images were produced using Helicon Focus 4.75 Pro from a series of source images taken by a Nikon EOS Kiss×4 digital camera attached to a Nikon ECLIPSE E600 microscope. Workers of each species were measured for the following parts using a micrometer, recorded to the second decimal place.

The abbreviations used for the measurements and indices are as follows:

CI Cephalic index, HW/HL × 100.

HL Maximum head length in full-face view, measured from the middle of anterior clypeal margin to the middle of the posterior margin of head.

HW Maximum head width in full-face view.

ML Mesosomal length measured from the point at which the pronotum meets the cervical shield to the posterior margin of metapleuron in profile.

PL Petiole length measured from the anterior margin of the peduncle to the posteriormost point of tergite.

SI Scape index, SL/HW × 100.

SL Scape length excluding the basal of constriction and condylar bulb.

TL Total length, roughly measured from the anterior margin of head to the tip of gaster in stretched specimens.

Abbreviations of the type depositories are as follows:

AMK Ant Museum, Faculty of Forestry, Kasetsart University, Thailand.

BMNH The Natural History Museum, London, U.K.

KKIC Kasetsart Kampaengsaen Insect collection, Thailand.

MCSN Museo Civico di Storia Naturale “Giacomo Doria”, Genova, Italy

MCZC Museum of Comparative Zoology, Cambridge, MA, U.S.A.

MHNG Muséum d’Histoire Naturelle, Geneva, Switzerland.

NIAST The National Institute of Agro-Environmental Sciences, Tsukuba, Japan.

SKYC SKY Collection at Kagoshima University, Japan.

THNHM Natural History Museum of the National Science Museum, Thailand.

USC University of San Carlos, Cebu City, The Philippines.

The general terminology in the worker caste of the ants follows Hölldobler and Wilson (1990), and Bolton (1994). For the important characters in the genus *Aenictus* used in this paper, see Jaitrong and Yamane (2011).

## Systematics

### Revision of the *Aenictus javanus* group

***Aenictus javanus* group**

**Diagnosis.** In the previous paper (Jaitrong and Yamane 2011) this species group was defined as follows: head in full-face view with occipital corner convex; occipital margin lacking collar; antenna 10-segmented; antennal scape short, extending only half length of head; anterior clypeal margin roundly convex bearing 6-10 denticles; mandible subtriangular, masticatory margin with 3 teeth including the large apical tooth; frontal carina short, not extending beyond the level of posterior margin of torulus; parafrontal ridge absent; mesosoma in profile with dorsal margin almost flat; dorsal face of mesosoma meeting with lateral face at a right angle; propodeal junction angulated; propodeal declivity encircled with a thin rim. Subpetiolar process developed and triangular or subrectangular.

Head and first gastral segment entirely smooth and shiny except base of gastral tergite I and sternite I with dense small punctures. Body reddish brown to yellowish brown; typhlatta spot absent.

**Remarks.** This is a group of relatively small ants measuring 1.38-3.40 mm in total length. It is similar to the *Aenictus piercei* group sensu Jaitrong and Yamane (2011) in terms of body size and coloration, but in the former the anterior clypeal margin has several denticles, while it lacks denticles in the latter.

A size variation exists among the specimens of single colonies. There is a general tendency that smaller specimens have a much weaker punctation, more elongate head and shorter antennal scape than larger specimens.

**Checklist of species**

*Aenictus doydeei* Jaitrong & Yamane, 2011

*Aenictus duengkaei* Jaitrong & Yamane, sp. n.

*Aenictus javanus* Emery, 1896

*Aenictus longinodus* Jaitrong & Yamane, sp. n.

*Aenictus nishimurai* Terayama & Kubota, 1993

*Aenictus piercei* Wheeler & Chapman, 1930

#### Key to species based on the worker caste

**Table:** 

1	Basal margin of mandible with a distinct denticle behind large basal tooth (Fig. 2D); smaller species (HW 0.25–0.38 mm) (E. Thailand)	*Aenictus duengkaei* sp. n.
–	Basal margin ofmandible lacking denticle (Figs 1A, 3A, 4A, 6A); larger species (HW 0.40–0.65 mm)	2
2	Declivity of propodeum almost flat, with blunt lateral carinae, but not demarcated basally by a transverse carina (Philippines) (Fig. 6F)	*Aenictus piercei* Wheeler & Chapman
–	Declivity of propodeum shallowly concave, and margined with a thin carina both laterally and basally (Figs 1D, 5C)	3
3	With head seen in profile occipital corner bluntly angulated (almost right-angled) (Java and Borneo) (Fig. 3D)	*Aenictus javanus* Emery
–	With head seen in profile occipital corner rounded (Figs 1E, 4D, 5B)	4
4	Petiole distinctly longer than high; postpetiole almost as long as petiole (S. Thailand) (Fig. 4B, D)	*Aenictus longinodus* sp. n.
–	Petiole almost as long as high; postpetiole slightly larger than petiole (Figs 1C, 5D, E)	5
5	Lateral face of pronotum shiny but with reticulation (Vietnam, Laos, and Thailand) (Fig. 1E)	*Aenictus doydeei* Jaitrong & Yamane
–	Lateral face of pronotum smooth and shiny; sculpture if any very superficial (Vietnam, Laos, and Thailand) (Fig. 5B)	*Aenictus nishimurai* Terayama & Kubota

#### Aenictus doydeei

Jaitrong & Yamane, 2011

http://species-id.net/wiki/Aenictus_doydeei

Figs 1 7A

Aenictus doydeei Jaitrong & Yamane, in Jaitrong et al. 2011: 319, figs 7–9.

##### Types.

Holotype and 61 paratype workers from a plantation, 211 m, Sivilay Village, Naxaythong Dist., Vientiane, Laos, 18°16'10"N, 102°26'36"E, 10.VI.2010, W. Jaitrong leg., WJT10-LAO13 (AMK, BMNH, KKIC, MCZC, SKYC, THNHM, examined).

##### Measurements.

Holotype: TL 3.40 mm; HL 0.70 mm; HW 0.65 mm; SL 0.40 mm; ML 1.00 mm; PL 0.28 mm; CI 93; SI 62.

Paratype workers(n= 9): TL 2.90–3.40 mm; HL 0.53–0.70 mm; HW 0.48–0.65 mm; SL 0.28–0.40 mm; ML 0.75–1.00 mm; PL 0.23–0.28 mm; CI 91–95; SI 55–62.

##### Description of worker

(holotype and paratypes).Head in full-face view almost as long as broad, with sides convex and posterior margin almost straight or feebly concave; seen in profile occipital corner of head rounded. Antennal scape reaching midlength of head; antennal segment II longer and narrower than each of III–VI; terminal segment (X) about 2.5 times as long as broad. Anterior margin of clypeus bearing 9–10 denticles. Masticatory margin of mandible with 3 acute teeth including a large apical tooth; basal margin lacking denticles. Mesosoma seen from above broader anteriorly than posteriorly; promesonotum laterally edged, in profile weakly convex dorsally and sloping gradually to propodeal junction; in profile propodeum slightly lower than promesonotum and almost flat dorsally; suture between mesopleuron and metapleuron completely absent; propodeal junction angulate, right-angled; declivity of propodeum shallowly concave, encircled by a thin rim. Petiole almost as long as high, its dorsal outline slightly elevated posteriorly; subpetiolar process well developed, subrectangular, its ventral margin almost straight and longer than posterior margin; postpetiole seen in profile subrectangular and slightly larger than petiole.

Head entirely smooth and shiny. Dorsal surface of pronotum smooth and shiny, lateral face superficially reticulate and shiny; mesothorax, metapleuron and propodeum densely microreticulate. Petiole entirely microreticulate. Postpetiole microreticulate except for a small smooth and shiny area on dorsal surface.

Head and mesosoma dorsally with relatively sparse standing hairs mixed with sparse short hairs; longest pronotal hairs 0.18–0.20 mm long. Head, mesosoma, petiole and postpetiole reddish brown; gaster yellowish brown; propodeum darker than other parts

**Figure 1.**
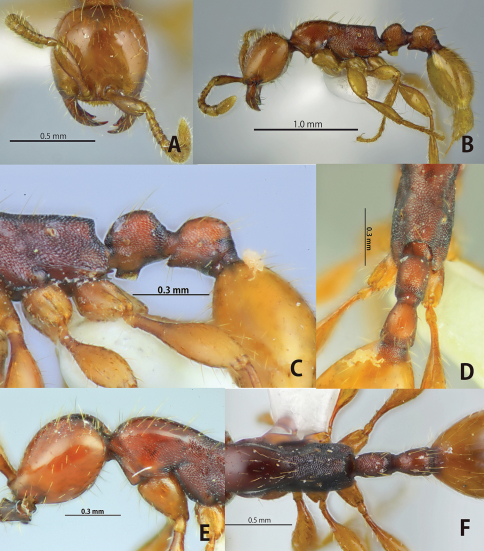
*Aenictus doydeei* (paratype). **A** Head in full-face view **B** habitus in profile **C** propodeal junction, petiole and postpetiole in profile **D** propodeal declivity with body in dorsal view **E** occipital corner of head in profile **F** dorsal view of body.

##### Non-type material examined.

**VIETNAM:** Dong Nai Prov., S. Cat Tien N.P., Crocodile Lake Trail, 18.X.2004, K. Eguchi leg., Eg04-VN-707 (SKYC). **THAILAND:** NE. Thailand, Loei Prov., Phu Rur Dist., disturbed area, 10.IV.2008, P. Kosolpanyapiwat leg., PKK08-TH102 (SKYC, THNHM); Chaiyaphum Prov., Phu Kheao Dist., Agricultural area, 30.I.1999, W. Jaitrong leg., WJT99-AG22 (SKYC, THNHM); NE. Thailand, Nakhon Ratchasima Prov., Sakaerat ERS, 10.VII.1999, Sk. Yamane leg., TH99-SKY-19 (SKYC, THNHM).

##### Distribution

**.** Vietnam (new record), Laos and Thailand (Fig. 7A).

##### Bionomics.

The type series from Laos and three colonies from Thailand were collected from disturbed areas in the night. Thus *Aenictus doydeei* is very probably nocturnal. Jaitrong et al. (2011) reported that this species preyed on *Pheidole plagiaria*.

##### Remarks.

This species is closely related to *Aenictus javanus*, *Aenictus longinodus*, and *Aenictus nishimurai* in terms of body size and coloration. *Aenictus doydeei*, however, is easily distinguished from *Aenictus javanus* and *Aenictus longinodus* as follows: occipital margin of head in profile rounded (Fig. 1E), while angled in *Aenictus javanus* (Fig. 3D); petiole almost as long as high, but clearly longer than high in *Aenictus javanus* and *Aenictus longinodus* (Figs 3B, 4B, D). *Aenictus doydeei* is most similar to *Aenictus nishimurai*, but is clearly larger than *Aenictus nishimurai* with a slight overlap, and has the lateral face of the pronotum that is smooth but reticulated (almost smooth in *Aenictus nishimurai*). *Aenictus doydeei* is sympatric with *Aenictus nishimurai* in Vientiane province, Laos and in northeastern Thailand.

#### Aenictus duengkaei

Jaitrong & Yamane sp. n.

urn:lsid:zoobank.org:act:DB41057D-3357-4A91-9A4A-25BCFA4926F8

http://species-id.net/wiki/Aenictus_duengkaei

Figs 2 7A

##### Types.

Holotype worker from E. Thailand, Chacheongsao Prov., Khao Ang Reu Nai, dry evergreen forest, 22.VIII.2003, Sk. Yamane leg., TH03-SKY-79 (THNHM). Twelve paratype workers, same data as holotype (BMHN, MCZC, SKYC, THNHM).

##### Measurements.

Holotype: TL 1.90 mm; HL 0.43 mm; HW 0.38 mm; SL 0.20 mm; ML 0.58 mm; PL 0.15 mm; CI 88; SI 53.

Larger workers(paratypes, n= 5): TL 1.80–1.90 mm; HL 0.43–0.45 mm; HW 0.36–0.38 mm; SL 0.19–0.20 mm; ML 0.53–0.58 mm; PL 0.14–0.15 mm; CI 83–88; SI 52–53. Smaller workers(paratypes, n= 3): TL 1.50–1.55 mm; HL 0.40–0.43 mm; HW 0.25–0.28 mm; SL 0.15–0.16 mm; ML 0.45–0.48 mm; PL 0.10–0.13 mm; CI 63–65; SI 59–60.

##### Description of worker

(holotype and paratypes)**.** Head in full-face view distinctly longer than broad and subrectangular, with sides weakly convex or almost parallel, and posterior margin clearly concave; seen in profile occipital corner of head rounded. Antennal scape very short, not reaching midlength of head; antennal segment II clearly longer than each of III-VI; III-VI shorter than broad; terminal segment longer than VII+VIII+IX and about 1.6 times as long as broad. Anterior margin of clypeus bearing 5–7 denticles. Masticatory margin of mandible with 3 acute teeth including a large apical tooth; basal margin with 1–2 denticles behind large basal tooth. Mesosoma seen in profile almost flat dorsally; propodeal junction angulate; declivity of propodeum almost flat, with blunt lateral carinae, but not demarcated basally by a transverse carina. Petiole round almost as long as high; subpetiolar process well developed, subrectangular, its ventral border almost straight and longer than posterior border; postpetiole slightly smaller than petiole and its dorsal outline roundly convex.

Head and antennal scape smooth and shiny; mandible extensively smooth but narrow zone along basal margin sculptured. Dorsal and lateral face of pronotum smooth and shiny except for anteriormost portion microreticulate; mesonotum smooth and shiny; mesopleuron superficially shagreened with smooth and shiny interspaces; metapleuron and propodeum shiny but microreticulate. Petiole entirely microreticulate but its dorsal face with a small area that is smooth (in larger specimens this area weakly sculptured). Postpetiolar node almost smooth and shiny.

Head with relatively sparse standing hairs; mesosoma dorsally with relatively dense standing hairs mixed with sparse short hairs over the surface; longest pronotal hairs 0.07–0.10 mm long. Head, gaster and legs yellowish brown; mesosoma, petiole and postpetiole reddish brown; mandible darker than elsewhere.

**Figure 2.**
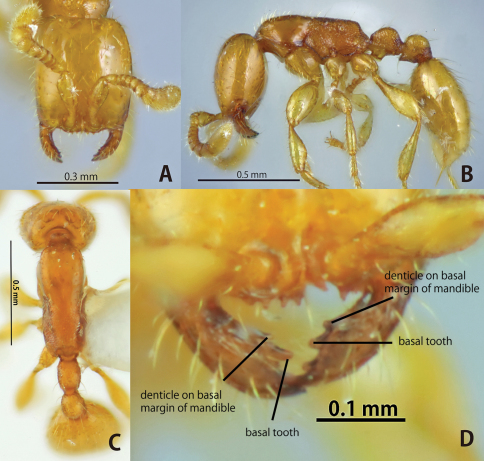
*Aenictus duengkaei* sp. n. (holotype). **A** Head in full-face view **B** habitus in profile **C** dorsal view of body; **D**, mandible and anterior clypeal margin.

##### Etymology.

The specific name is dedicated to Dr. Prateep Duengkae of the Faculty of Forestry, Kasetsart University, who helped us in collecting material in eastern Thailand.

##### Non-type material examined.

**THAILAND:** E. Thailand, Chonburi Prov., Kasetsart Siracha campus, agriculture area, 20.III.2004, Wanishsakulpong leg., WJT04-E50 (THNHM).

##### Distribution.

E. Thailand (Fig. 7A).

##### Bionomics.

This species has been known only from eastern Thailand. The type series was collected from soil in a lowland dry evergreen forest (ca. 200 m), while the other colony (WJT04-E50) was collected from soil in an agricultural area. Thus, this species inhabits both primary and disturbed forests.

##### Remarks.

*Aenictus duengkaei* is similar to *Aenictus piercei* in terms of body size and coloration. Furthermore, the propodeal declivity is not margined basally with a carina in both species. However, *Aenictus duengkaei* is easily separated from the latter by the condition of the mandible that has a distinct denticle on the basal margin, while the denticle is lacking in *Aenictus piercei*.

#### Aenictus javanus

Emery, 1896

http://species-id.net/wiki/Aenictus_javanus

Figs 3 7B

Aenictus javanus Emery 1896: 245; Forel 1909: 222; Wilson 1964: 467 figs 36; Bolton 1995: 59.

##### Types.

Two syntype males from Java, Buitenzorg [Bogor] (MCSN, examined).

##### Measurements.

Non-type workersfrom the type locality (n= 8): TL 2.35–2.60 mm; HL 0.55–0.58 mm; HW 0.50–0.53 mm; SL 0.35 mm; ML 0.83–0.88 mm; PL 0.23–0.25 mm; CI 91; SI 67–70.

##### Description of worker

(non-type workers from the type locality)**.** Head in full-face view slightly longer than broad, subrectangular, with sides convex and posterior margin almost straight or feebly concave; seen in profile occipital corner of head angulated. Antennal scape reaching midlength of head; antennal segment II almost as long as each of III-V; terminal segment longer than VII+VIII+IX and 2.2 times as long as broad. Anterior margin of clypeus bearing 6–7 denticles. Masticatory margin of mandible with 3 acute teeth including a large apical tooth; basal margin lacking denticles. Promesonotum in profile weakly convex dorsally or almost flat and sloping gradually to propodeal junction; in profile propodeum almost flat dorsally; suture between mesopleuron and metapleuron almost absent; propodeal junction angulate, right-angled; declivity of propodeum shallowly concave, encircled by a thin rim. Petiole distinctly longer than high, its dorsal outline slightly elevated posteriorly; subpetiolar process well developed, subrectangular, its ventral border almost straight or feebly concave and as long as posterior border; postpetiole almost as long as, its dorsal outline slightly convex.

Head including antennal scape smooth and shiny; mandible striate along basal margin and smooth in apical and peripheral parts. Dorsal surface of pronotum smooth and shiny, lateral face of pronotum superficially reticulate but shiny; anteriormost part of pronotum microreticulate; mesothorax, metapleuron and propodeum microreticulate. Petiole entirely microreticulate. Postpetiole microreticulate except for a small area on dorsal surface smooth and shiny.

Head and mesosoma dorsally with relatively sparse standing hairs mixed with sparse short hairs; longest pronotal hairs 0.15–0.18 mm long. Head yellowish brown to redish brown, mesosoma, petiole and postpetiole reddish brown; gaster yellowish brown. Typhlatta spot absent.

**Figure 3.**
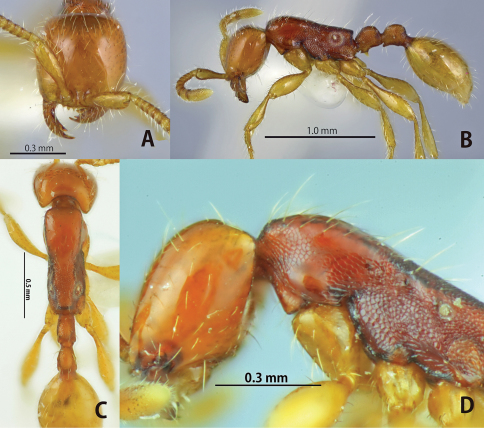
*Aenictus javanus* (non-type from Java). **A** Head in full-face view **B** habitus in profile **C** dorsal view of body **D** occipital corner of head.

##### Non-type material examined.

**MALAYSIA:** Borneo, Sabah, Sandakan, Sepilok, Water Hole Trail, 30.V.2005, Alveron leg., A46 (SKYC); Borneo, Sabah, Sepilok forest, 27.I.1997, K. Eguchi leg., Eg97-BOR-506; Borneo, Sarawak, Lambir Hills N.P., 8 ha Plot, 11.VII.2004, H.O. Tanaka leg., TY04–801 (SKYC, THNHM). **BRUNEI:** Temburong, Kuala Belalong, Field Studies Centre, 19.II.1999, K. Eguchi leg., Eg97-BOR-225 (SKYC, THNHM). **INDONESIA:** W. Java, Bogor, Kebun Raya, 9.XII.1995, F. Ito leg., FI95–536 (SKYC, THNHM); same loc., 25.II.1997, F. Ito leg., FI97–06 (SKYC, THNHM).

Six workers from Java (2 pins, three on each pin, labeled as typus) identified as *Aenictus javanus* by Auguste-Henri Forel (MHNG) were examined. This series should be the same as that cited in Forel (1909). These workers are not the types.

##### Distribution.

Borneo (Sabah, Sarawak, and Brunei) and Java (Bogor) (Fig. 7B).

##### Bionomics.

All colonies of this species were collected from lowland rainforests.

##### Remarks.

This species is closely related to *Aenictus doydeei*, *Aenictus longinodus*, and *Aenictus nishimurai* in terms of body size and coloration. Among these species is more closely related to *Aenictus longinodus* than the others in having the long petiole. *Aenictus javanus* can be separated from *Aenictus longinodus* as follows: occipital margin of head in profile angulated, while rounded in *Aenictus longinodus*; the lateral face of the pronotum that are reticulate but shiny (almost smooth in *Aenictus longinodus*).

#### Aenictus longinodus

Jaitrong & Yamane sp. n.

urn:lsid:zoobank.org:act:69B819C3-94CE-47A3-8B62-3291A6BA2572

http://species-id.net/wiki/Aenictus_longinodus

Figs 4 7B

##### Types.

Holotype worker from S. Thailand, Trang Prov., Khao Chong Botanical Garden, evergreen forest, 8.XI.2003, W. Jaitrong, THNHM-I03–942 (= TH03-WJT-713, THNHM). Thirty-seven paratype workers, same data as holotype (BMHN, MCZC, MHNG, SKYC, THNHM).

##### Measurements.

Holotype: TL 2.55 mm; HL 0.55 mm; HW 0.53 mm; SL 0.33 mm; ML 0.80 mm; PL 0.25 mm; CI 95; SI 62.

Larger workers(paratypes, n= 8): TL 2.45–2.60 mm; HL 0.55–0.58 mm; HW 0.53–0.55 mm; SL 0.33–0.35 mm; ML 0.80–0.88 mm; PL 0.25 mm; CI 95–96; SI 61–64. Smaller workers(paratypes, n= 4): TL 1.90–2.00 mm; HL 0.45–0.53 mm; HW 0.40–0.43 mm; SL 0.22–0.28 mm; ML 0.63–0.73 mm; PL 0.18–0.19 mm; CI 85–89; SI 56–61.

##### Description of worker

(holotype and paratypes)**.** Head in full-face view slightly longer than broad, with sides convex and posterior margin almost straight; seen in profile occipital corner of head rounded. Antennal scape reaching midlength of head; antennal segment II longer and narrower than each of III-VI; terminal segment about 2 times as long as broad. Anterior margin of clypeus bearing 7 denticles. Masticatory margin of mandible with 3 acute teeth including a large apical tooth; basal margin lacking denticles. Promesonotum seen in profile almost flat or weakly convex dorsally and sloping gradually to propodeal junction; in profile propodeum slightly lower than promesonotum and almost flat dorsally; propodeal junction angulate, right-angled; declivity of propodeum shallowly concave, encircled by a thin rim. Petiole longer than high, its dorsal outline convex; subpetiolar process well developed, subrectangular, its ventral margin almost straight or weakly convex and slightly longer than posterior margin; postpetiole seen in profile subrectangular and slightly shorter than petiole.

Head including antennal scape smooth and shiny; mandible striate along basal margin and smooth in apical and peripheral parts. Dorsal and lateral surface of pronotum smooth and shiny except for anteriormost part microreticulate; mesothorax, metapleuron and propodeum microreticulate. Petiole entirely microreticulate. Postpetiole microreticulate except for a small smooth and shiny area on dorsal surface.

Head and mesosoma dorsally with relatively sparse standing hairs mixed with sparse short hairs over the surface; longest pronotal hairs 0.13–0.15 mm long. Head, mesosoma, petiole and postpetiole reddish brown; gaster yellowish brown and paler than the other parts of body; propodeum darkest.

**Figure 4.**
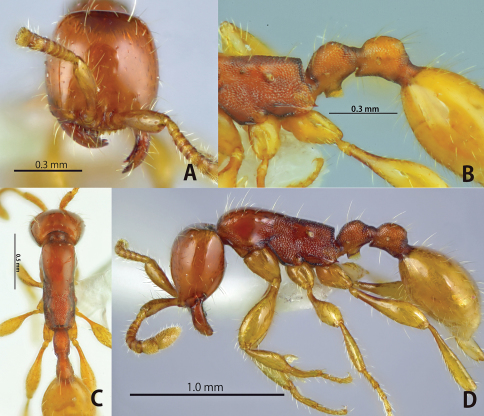
*Aenictus longinodus* sp. n. (paratype). **A** Head in full-face view **B** propodeal junction, petiole and postpetiole in profile **C** dorsal view of body **D** habitus in profile.

##### Etymology.

The specific name refers to the long petiole.

##### Non-type material examined.

**THAILAND:** S. Thailand, Nakhon Si Thammarat Prov., Nuppitam Dist., Khao Luang N.P., Krung Ching waterfall, rainforest, 20.V.2003, W. Jaitrong leg., THTHM-I03–3779 (= TH03-WJT330, THNHM); Trang Prov., Palian Dist., Ban Suso, Open area, 31.X.2011, W. Jaitrong leg., TH11-WJT-183 (SKYC, THNHM); Songkhla Prov., Ton Nga Chang W.S., 5.II.1998, N. Noon-anant leg., N98–3 (SKYC, THNHM); same loc., 29.X.1997, N. Noon-anant leg., N97–1 (SKYC, THNHM); Songkhla Prov., Hat Yai, Songkhlanakarin Campus, PSU forest, 21.X.2011, Sk. Yamane leg., TH11-SKY-166 (SKYC, THNHM).

##### Distribution.

Malay Peninsula (S. Thailand) (Fig. 7B).

##### Bionomics.

The type series, two colonies from Ton Nga Chang Wildlife Sanctuary (N98–3 and N97–1), and a colony from Khao Luang National Park (TH03-WJT330) were collected in lowland rainforests. A colony (TH11-WJT-183) was collected from an open area in the day, while a colony (TH11-SKY-166), just coming out of soil, was from a disturbed forest near a concrete road in the night. Thus, this species inhabits both primary and disturbed forests and is active in the day and night.

##### Remarks.

This species is most similar to *Aenictus javanus*. See under *Aenictus javanus* for details.

#### Aenictus nishimurai

Terayama & Kubota, 1993

http://species-id.net/wiki/Aenictus_nishimurai

Figs 5 7A

Aenictus nishimurai Terayama and Kubota 1993: 70, figs 9–10; Jaitrong et al. 2011: 321, figs 10–12.

##### Types.

Holotype and 10 paratype workers (NIAST, SKYC) from Thailand, Changmai Prov. [Chiangmai Prov.], Doi Suthep (1,500 m alt.), 18.VIII.1992, M. Terayama and S. Kubota leg. A paratype in SKYC was examined.

##### Measurements.

Paratype: TL 2.40 mm; HL 0.58 mm; HW 0.48 mm; SL 0.25 mm; ML 0.75 mm; PL 0.20 mm; CI 83; SI 53.

Larger workers(non-types, n= 7): TL 2.66–2.90 mm; HL 0.60–0.65 mm; HW 0.53–0.58 mm; SL 0.33–0.35 mm; ML 0.83–0.90 mm; PL 0.23–0.25 mm; CI 88; SI 61–64. Smaller workers(non-types, n= 4): TL 1.95–2.25 mm; HL 0.48–0.50 mm; HW 0.38–0.43 mm; SL 0.20–0.25 mm; ML 0.55–0.65 mm; PL 0.15–0.18 mm; CI 79–85; SI 53–59.

##### Description of worker

(paratype and non-type workers)**.** Head in full-face view longer than broad, with sides slightly convex and posterior margin almost straight or feebly concave; seen in profile occipital corner of head rounded. Antennal scape reaching midlength of head; antennal segment II almost as long as broad; III-VIII each slightly broader than long; terminal segment 2.3 times as long as broad. Anterior margin of clypeus bearing 7–10 denticles. Masticatory margin of mandible with 3 acute teeth including a large apical tooth; basal margin lacking denticles. Mesosoma seen in profile weakly convex dorsally or almost flat; in profile propodeum almost flat dorsally; suture between mesopleuron and metapleuron absent; propodeal junction dully angulated, forming an almost right angle; declivity of propodeum shallowly concave, encircled by a thin rim. Petiole nearly as long as high, its dorsal outline convex; subpetiolar process well developed, subrectangular, its ventral margin nearly straight and longer than posterior margin; postpetiole seen in profile almost as long as petiole, with round node.

Head including antennal scape entirely smooth and shiny; mandible finely striate with outer zone smooth and shiny. Dorsal and lateral surface of pronotum smooth and shiny except for anteriormost part microreticulate; mesothorax, metapleuron, and propodeum microreticulate. Petiole entirely microreticulate. Postpetiole microreticulate except for smooth and shiny area on dorsal surface.

Head and mesosoma dorsally with relatively sparse standing hairs mixed with sparse short hairs; longest pronotal hairs 0.15–0.18 mm. Head yellowish brown; mesosoma, petiole and postpetiole reddish brown; gaster yellowish brown, but paler than head.

**Figure 5.**
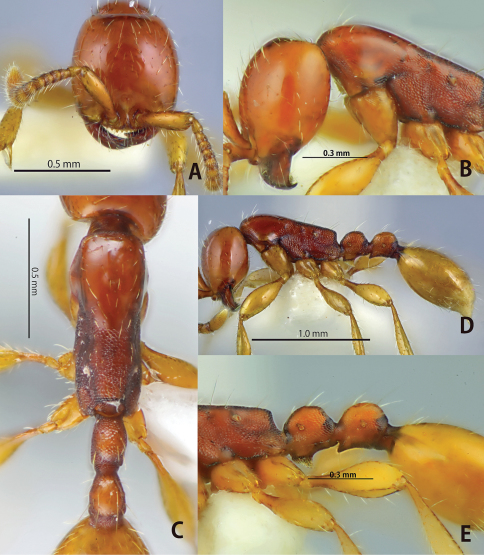
*Aenictus nishimurai* (non-type from Chiang Mai Province, N. Thailand). **A** Head in full-face view **B** head in profile showing occipital margin **C** dorsal view of body **D** habitus in profile **E** propodeal junction, petiole and postpetiole in profile.

##### Non-type material examined.

**VIETNAM:** N. Vietnam, Ha Tai Prov., Ba Vi N.P., 400–600 m alt., 12.XI.1999, K. Eguchi leg., Eg99-VN-107 (SKYC, THNHM); same loc., 11.XI.1999, K. Eguchi leg., Eg99-VN-84 (SKYC, THNHM); Bac Giang, Tay Yen Tu, 400 m alt., 23.V.2004, K. Eguchi leg., Eg04-VN-100 (SKYC). **LAOS:** Vientiane Prov., Pak Ngum Dist., Phang Dang Village, 14.VI.2010, W. Jaitrong leg., WJT10-LAO19, Sk. Yamane leg., LA10-SKY-56 (AMK, SKYC, THNHM). **THAILAND:** N. Thailand, Chiang Mai Prov., Mae Tang dist., 26.IV.2000, W. Jaitrong leg., WJT00-TH01 (SKYC, THNHM); W. Thailand, Kachanaburi Prov., Sai Yok N.P., 140 m alt., 30.VI.2002, Sk. Yamane leg., TH02-SKY-19 (SKYC, THNHM); NE. Thailand, Saraburi Prov., Phukae B.G., 1.VII.2002, Sk. Yamane leg., TH02-SKY-41 (SKYC, THNHM).

##### Distribution.

Vietnam, Laos and Thailand.

##### Bionomics.

No biological information is available for *Aenictus nishimurai*. However, judging from the localities cited above this species is distributed from lowland to highland (200–1,500 m) and inhabits primary, secondary and disturbed forests.

##### Remarks.

This species is most similar to *Aenictus doydeei*. See under *Aenictus doydeei* for details.

#### Aenictus piercei

Wheeler & Chapman, 1930

http://species-id.net/wiki/Aenictus_piercei

Figs 6 7B

Aenictus piercei Wheeler & Chapman, in Wheeler 1930: 209, fig. 7e–g; Wilson 1964: 474, figs 61–62; Bolton 1995: 60.

##### Types.

**Figure 6.**
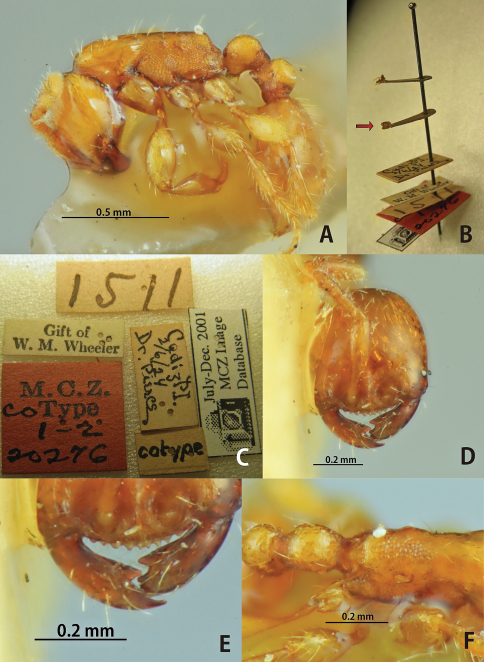
*Aenictus piercei* (A, B, C, F, lectotype; D, E, non-type from the type locality). **A** Habitus in profile **B** lectotype and paralectotype designated in the present paper (arrow indicating the lectotype) **C** labels of lectotype **D** head in full-face view **E** mandible and anterior clypeal margin **F** propodeal declivity in dorsal view.

Two syntype workers on a pin, the Philippines, Negros, Cadiz, 2.VI.1924, leg. Dr. Pierce (MCZC, examined). The worker located below on the pin is selected as the lectotype (Fig. 6B).

##### Measurements.

Lectotype: TL 2.15 mm; HL 0.53 mm; HW 0.48 mm; SL 0.28 mm; ML 0.70 mm; PL 0.20 mm; CI 90; SI 58.

Paralectotypes (n = 2): TL 2.15 mm; HL 0.53–0.58 mm; HW 0.48–0.50 mm; SL 0.28–0.33 mm; ML 0.70–0.83 mm; PL 0.20–0.23 mm; CI 87–90; SI 58–65.

##### Description of worker

(lectotype, paralectotype and a non-type worker)**.** Head in full-face view slightly longer than broad, subrectangular, with sides feebly convex and posterior margin almost straight; seen in profile occipital corner of head rounded. Antennal scape reaching midlength of head; antennal segment II longer and narrower than each of III–VI; terminal segment almost as long as VII+VIII+IX and 1.9 times as long as broad. Anterior margin of clypeus bearing 9–10 denticles (this observation is based on the single non-type worker, since in the lectotype mouth parts are buried in glue and the head of the paralectotype was missing). Masticatory margin of mandible with 3 acute teeth including large apical tooth; basal margin lacking denticles. Promesonotum in profile almost flat dorsally; in profile propodeum almost flat dorsally; propodeal junction angulate, right-angled; declivity of propodeum nearly flat, with blunt lateral carinae, but not demarcated basally by a transverse carina. Petiole almost as long as high, its dorsal outline convex; subpetiolar process well developed, subrectangular, its ventral margin slightly convex and longer than posterior margin; postpetiole almost as long as petiole.

Head including antennal scape entirely smooth and shiny. Mandible finely striate except along masticatory and outer margins. Pronotum entirely smooth and shiny except for anteriormost part microreticulate; mesonotum smooth and shiny; mesopleuron, metapleuron and propodeum microreticulate. Petiole entirely microreticulate. Postpetiole microreticulate except for a small smooth and shiny area on dorsal surface.

Head and mesosoma dorsally with relatively sparse standing hairs mixed with sparse short hairs; longest pronotal hairs 0.09–0.10 mm long. Entire body yellowish brown or reddish brown; legs palest.

##### Non-type material examined.

We examined a worker collected from the same place by Chapman but in a different year (2/6/29). It bears a small piece of white paper with handwriting “cotype”, and a small piece of red paper. As this specimen was not mentioned in the original description, it is not part of the type series. However, all the three specimens belong to the same species without doubt.

##### Distribution.

**Figure 7.**
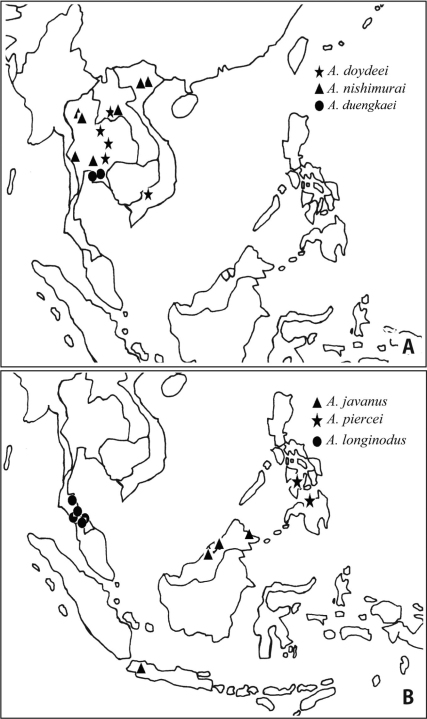
Distribution of the species of the *Aenictus javanus* group. **A** *Aenictus doydeei*, *Aenictus nishimurai*, and *Aenictus duengkaei* sp. n. **B** *Aenictus javanus*, *Aenictus longinodus* sp. n., and *Aenictus piercei*.

Philippines (Negros and Mindanao) (Fig. 7B).

##### Bionomics.

Little is known about the bionomics of *Aenictus piercei*. Nothing is mentioned by Wheeler (1930) on it. However, judging from the hitherto known localities (Negros and Mindanao) (see Wilson 1964) this species inhabits lowland (15–600 m) and is probably restricted to the Philippines. Wilson (1964) cited India, Solon (ca. 1400 m), as a locality of *Aenictus piercei*, but the identification should be reconfirmed.

##### Remarks.

This species is most similar to *Aenictus duengkaei* (see under *Aenictus duengkaei* for differences). According to Wilson (1964) the clypeus of *Aenictus piercei* has an entire anterior margin without denticles. Following this information, Jaitrong and Yamane (2011) treated *Aenictus piercei* as a member of their *Aenictus piercei* group (no denticles in this group). However, the non-type specimen mentioned above has nine denticles on the anterior clypeal margin. After carefully examining the type material of *Aenictus piercei* we concluded that this species should be removed from the *Aenictus piercei* group and that it is a member of the *Aenictus javanus* group.

### Revision of the *Aenictus philippinensis* group

***Aenictus philippinensis* group**

**Diagnosis.** Jaitrong and Yamane (2011) defined this species group as follows: antenna 10-segmented; scape not reaching the posterolateral corner of head; anterior clypeal margin convex in the middle, lacking denticles; mandible triangular, very densely with punctures; its masticatory margin with a large and sharp apical tooth followed by 6–8 small inconspicuous denticles; basal margin lacking denticles; frontal carinae fused at the level of antennal base to form a single carina, extending less than half length of head, and well developed anteriorlly and poorly developed posteriorly; parafrontal ridge present, not reaching midlength of head; occipital margin forming a collar or carina; mesosoma in profile with promesonotum convex dorsally and sloping gradually to metanotal groove; mesopleuron clearly demarcated from metapleuron by a deep groove and from promesonotum by a distinct carina; metanotal groove relatively deep and distinct; propodeal junction angulated; declivity of propodeum concave, encircled with a rim; subpetiolar process weakly developed.

First gastral segment entirely smooth and shiny except the base of both tergite and sternite with dense small punctures. Body reddish brown to dark brown; typhlatta spot absent.

**Remarks.** This group consists of relatively large species measuring 4.05–4.60 mm in total body length, and is closely related to the *Aenictus pachycerus* group and *Aenictus hottai* group.However, the *Aenictus philippinensis* group is separated from the other two by the mesonotum demarcated from the mesopleuron by a conspicuous ridge and the metanotal groove relatively deep and distinct. The sculpture of the head is variable, from entirely smooth to densely puncto-reticulate (see Jaitrong and Yamane 2011).

Worker caste is clearly monomorphic.

**Check list of species**

*Aenictus pangantihoni* Zettel & Sorger, 2010

*Aenictus philippinensis* Chapman, 1963

*Aenictus punctatus* Jaitrong & Yamane, sp. n.

*Aenictus rabori* Chapman, 1963

#### Key to species based on the worker caste

**Table:** 

1	Frons of head smooth and shiny; mandible extensively smooth and shiny, with scattered punctures, or striae confined to periphery	2
–	Frons of head sculptured (superficially to very densely punctate); mandible almost entirely sculptured.	3
2	With head seen in profile occipital corner produced as a small lobe (Fig. 11C); sides of head partly superficially shagreened with smooth and shiny interspaces; larger species (HW 0.83–0.85 mm; TL 4.35–4.45 mm)	*Aenictus rabori* Chapman
–	With head seen in profile occipital corner without such a lobe (Figs 8B, 9B, 10C); sides of head entirely smooth and shiny; smaller species (HW 0.78–0.80 mm; TL 4.00–4.10 mm)	*Aenictus pangantihoni* Zettel & Sorger
3	Head entirely punctate, punctures fine and very dense; dorsal face of pronotum punctate (Borneo and Java)	*Aenictus punctatus* Jaitrong & Yamane, sp. n.
–	Head superficially reticulate, slightly shiny; dorsal face of pronotum almost smooth and shiny (Philippines)	*Aenictus philippinensi*s Chapman

#### Aenictus pangantihoni

Zettel & Sorger, 2010

http://species-id.net/wiki/Aenictus_pangantihoni

Figs 8 12

Aenictus pangantihoni Zettel and Sorger 2010: 120, figs. 5–8, 13.

##### Types.

Holotype (USC) and 56 paratype workers (NHMV, SKYC, THNHM) from Philippines, Camiguin, West of Mambajao, Katibawasan area, 350 m a.s.l., H. Zettel and C.V. Pangantihon leg. Four paratype workers in SKYC and THNHM were examined.

##### Measurements.

Paratype (n = 4): TL 4.00–4.10 mm; HL 0.83–0.88 mm; HW 0.78–0.80 mm; SL 0.55–0.63 mm; ML 1.38–1.43 mm; PL 0.35–0.38 mm; CI 91–95; SI 75–78.

##### Description of worker

(paratypes)**.** Head in full-face view slightly longer than broad, with sides slightly convex and posterior margin almost straight; occipital margin forming a distinct carina; seen in profile occipital corner of head rounded. Antennal scape relatively short, slightly extending 2/3 of head length; antennal segment II slightly longer than each of III-VI; terminal segment almost as long as VII+VIII+IX. Frontal carinae short fused at the level of antennal base to form a single carina and slightly extending beyond the level of the posterior margin of torulus, poorly developed in posterior half. Parafrontal ridge short, extending less than 1/3 of head length, 0.17 mm long, seen in profile weakly developed in the middle. Masticatory margin of mandible with a large apical tooth followed by a series of 7–9 denticles of two sizes, the larger ones alternating with 1–3 of smaller size. Mesosoma in profile with promesonotum weakly convex dorsally and sloping gradually to metanotal groove; metanotal groove distinct and deep; upper portion of mesopleuron impressed; propodeum slightly lower than mesonotum; propodeal junction right-angled; declivity of propodeum shallowly concave, encircled with a distinct rim. Petiole subsessile, distinctly longer than high; subpetiolar process almost absent; postpetiole as long as petiole (including short pedicel) and almost as long as high, with its node rounded dorsally. Legs relatively short, seen from side with greatly swollen femora.

Head entirely smooth and shiny, except for hair pits; mandible smooth and shiny, with scattered punctures; antennal scape superficially shagreened. Pronotum smooth and shiny except for its anteriormost portion reticulate; lateral face of pronotum smooth and shiny, with a narrow ventral belt that is impressed and reticulate, this belt continuing posteriorly, running along posterior margin of the lateral face, approaching dorsal face of pronotum; mesonotum smooth and shiny; mesopleuron, metapleuron and propodeum densely punctuate/reticulate and mat except for isolated small shiny areas. Both petiole and postpetiole microrecticulate except dorsal faces smooth and shiny. Femora superficially shagreened with smooth and shiny interspaces; tibiae superficially shagreened, partly smooth and shiny.

Head and mesosoma dorsally with relatively sparse standing hairs; longest pronotal hair 0.2–0.25 mm long. Entire body reddish brown.

**Figure 8.**
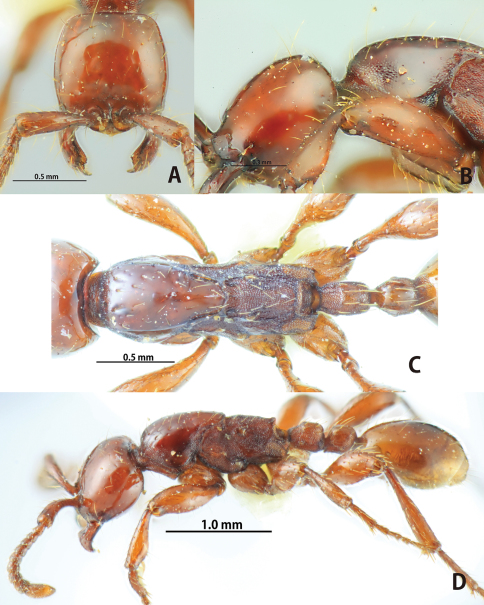
*Aenictus pangantihoni* (paratype). **A** Head in full-face view **B** occipital corner of head **C** dorsal view of body **D** habitus in profile.

##### Distribution.

Philippines (Camiguin Island) (Fig. 12).

##### Bionomics.

So far *Aenictus pangantihoni* is known only from the type locality. The type material was collected from a trail lined with some bushes and trees in a pasture area at an elevation ca. 350 m (Zettel and Sorger 2010).

##### Remarks.

*Aenictus pangantihoni* is most similar in general appearance to *Aenictus rabori*. However, it is easily distinguished from the latter as follows: smaller than *Aenictus rabori* (HW 0.78–0.80 mm, TL 4.00–4.10 mm in *Aenictus pangantihoni*; HW 0.83–0.85 mm, TL 4.35–4.45 mm in *Aenictus rabori*); seen in profile occipital corner of head round, without protruding lobe (with a lobe in *Aenictus rabori*); sides of head entirely smooth and shiny (partly superficially shagreened with smooth and shiny interspaces in *Aenictus rabori*).

#### Aenictus philippinensis

Chapman, 1963

http://species-id.net/wiki/Aenictus_philippinensis

Figs 9 12

Aenictus philippinensis Chapman 1963: 247, fig. 2.

##### Types.

Syntype workers from Philippines, Negros, Horns of Negros, 450 and 1,080 m (MCZC). We did not examine the type material of this species but specimens of a single colony from the type locality (Philippines, Negros) were examined.

##### Measurements.

Non-type workers (n = 10): TL 3.70–4.00 mm; HL 0.83–0.88 mm; HW 0.74–0.80 mm; SL 0.55–0.60 mm; ML 1.18–1.25 mm; PL 0.26–0.33 mm; CI 89–91; SI 74–77.

##### Description of worker.

Head in full-face view subretangular, slightly longer than broad, with sides weakly convex and posterior margin almost straight; occipital margin forming a narrow carina; seen in profile occipital corner of head rounded. Antennal scape relatively short, reaching only 2/3 of head length; antennal segment II almost as long as each of III-VI; terminal segment almost as long as VII+VIII+IX. Frontal carinae fused at the level of antennal base to form a single carina and extending beyond the level of the posterior margin of torulus, poorly developed in posterior half. Parafrontal ridge relatively long, extending less than 1/3 of head length, 0.25–0.28 mm long. Masticatory margin of mandible with a large apical tooth followed by a series of 6–7 denticles of same size. Mesosoma in profile with dorsally convex promesonotum and sloping gradually to metanotal groove; metanotal groove distinct and deep; mesopleuron relatively short, clearly dermacated from metapleuron by a deep groove; propodeum lower than mesonotum, weakly convex dorsally; propodeal junction right-angled; declivity of propodeum shallowly concave, encircled with a distinct rim. Petiole subsessile, slightly longer than high; subpetiolar process very low, its anteroventral corner bluntly angulate; postpetiole slightly longer than petiole and slightly longer than high, with its dorsal outline convex. Legs relatively long with apical halves of femora and tibiae somewhat swollen.

Head superficially reticulate and shiny; mandible very finely striate except along masticatory margin; antennal scape superficially shagreened. Promesonotum finely macroreticulate except dorsal face largely smooth and shiny; mesopleuron, metapleuron, and propodeum densely punctate/reticulate. Both petiole and postpetiole punctate except dorsal face of the latter smooth and shiny. Femora entirely superficially reticulate and shiny, partly smooth and shiny; tibiae weakly punctate.

Head and mesosoma dorsally with relatively sparse standing hairs mixed with short hairs over surface; longest pronotal hair 0.17–0.20 mm long. Entire body reddish brown.

**Figure 9.**
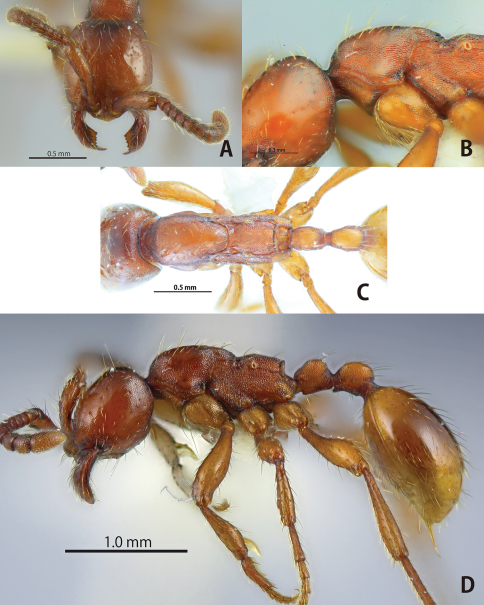
*Aenictus philippinensis* (non-type specimen from the Philippines). **A** Head in full-face view **B** occipital corner of head **C** dorsal view of body **D** habitus in profile.

##### Non-type material examined.

**Philippines:** Negros Oriental, near Dumaguete, Apolong, Valencia, 26.XII.1998, Sk. Yamane leg., PH98-SKY-05 (SKYC, THNHM).

##### Distribution.

Philippines (Negros) (Fig. 12).

##### Bionomics.

*Aenictus philippinensis* is very probably restricted to the Philippines and probably sympatric with *Aenictus rabori* in at least Negros Oriental. Wilson (1964) cited the observation by Chapman: “the workers of a colony came from the hole in the ground, climbed up a nearby stump, and spent the next hour in which they were observed building a living pyramid in the center of the stump. Some tried to build out from the edge of the stump in a horizontal direction.” We found a colony under a stone near a road.

##### Remarks.

*Aenictus philippinensis* is similar to *Aenictus punctatus* as they have sculptured head and mandible. However, they differ in some characters. The sculpturing on the head is much weaker in *Aenictus philippinensis* (superficially reticulate and shiny) than in *Aenictus punctatus* (finely punctate). Pronotal dorsum is smooth and shiny in *Aenictus philippinensis*, but finely punctate in *Aenictus punctatus*. Propodeal declivity is dorsally margined with a low rim in *Aenictus philippinensis*; the rim is much more developed, in profile distinctly protruding posteriad in *Aenictus punctatus*.

#### Aenictus punctatus

Jaitrong & Yamane sp. n.

urn:lsid:zoobank.org:act:7D3C938A-9108-421B-8ED1-4AF0AE4DB335

http://species-id.net/wiki/Aenictus_punctatus

Figs 10 12

##### Types.

Holotype from Brunei, Tasek Merimbun, 13.II.1999, K. Eguchi leg. Eg99-BOR-078 (SKYC). Nineteen paratype workers, same data as holotype (BMNH, MCZC, MHNG, SKYC, THNHM).

##### Measurements.

Holotype: TL 4.50 mm; HL 0.98 mm; HW 0.85 mm; SL 0.70 mm; ML 1.38 mm; PL 0.33 mm; CI 87; SI 82.

Paratypes (n = 9): TL 4.30–4.40 mm; HL 0.95–0.98 mm; HW 0.83–0.85 mm; SL 0.68–0.73 mm; ML 1.33–1.35 mm; PL 0.28–0.30 mm; CI 87; SI 82–85.

##### Description of worker

(holotype and paratypes)**.** Head in full-face view elliptical, clearly longer than broad, with sides convex and posterior margin almost straight or weakly convex; occipital margin forming a distinct carina; seen in profile occipital corner of head rounded. Antennal scape relatively long, extending 3/4 of head length; antennal segment II almost as long as each of III-VI; terminal segment slightly shorter than VII+VIII+IX. Frontal carinae short fused at the level of antennal base to form a single carina and slightly extending beyond 1/4 of head length, poorly developed in posterior half. Parafrontal ridge short, extending less than 1/3 of head length, 0.30–0.33 mm long. Masticatory margin of mandible with a series of 6–7 denticles of same size; basal margin of mandible lacking denticles. Mesosoma in profile with promesonotum convex dorsally and sloping gradually to metanotal groove; metanotal groove indistinct compared with those of the other members of the group; mesonotum demarcated from mesopleuron by a conspicuous ridge. Propodeum almost flat or weakly convex dorsally; declivity of propodeum shallowly concave, encircled with a developed rim; seen in profile dorsal portion of the rim protruding posteriad. Petiole subsessile, slightly longer than high, its dorsal outline elevated posteriorlly; subpetiolar process very low, its ventral outline weakly convex; postpetiole longer and larger than petiole and slightly longer than high, with its dorsal outline convex. Legs relatively long with apical halves of femora and tibiae somewhat swollen.

Head entirely finely punctate; mandible very finely striate except along masticatory margin; antennal scape finely punctate. Pronotum entirely punctate; mesopleuron, metapleuron and lateral face of propodeum punctate; dorsal face of propodeum finely punctate. Petiole entirely punctate; postpetiole punctate with weakly sculptured and shiny anterior slope of node. Basal half of femora densely punctate but apical half superficially macroreticulate and shiny; tibiae macroreticulate and shiny.

Head and mesosoma dorsally with sparse standing hairs mixed with very short hairs; longest pronotal hair 0.25–0.28 mm long. Entire body dark reddish brown. Typhlatta spots absent.

**Figure 10.**
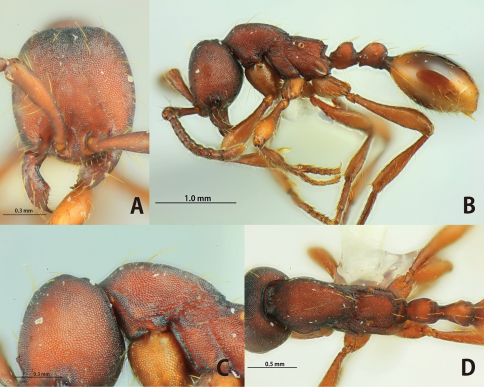
*Aenictus punctatus* **sp. n.** (holotype). **A** Head in full-face view **B** habitus in profile **C** occipital corner of head **D** dorsal view of body.

##### Etymology.

The species epithet “punctatus” is a Latin word meaning punctate. This refers to the finely punctate head of this species, while the head is reticulate or smooth and shiny in the other species of the *Aenictus philippinensis* group.

##### Non-type materials examined.

**Malaysia:** Borneo, Sabah, Sepilok Forest, 27.VIII.1995, Sk. Yamane leg. (SKYC, THNHM); Borneo, Sabah, Danum Valley, 4.XII.1996, K. Eguchi leg., Eg96-BOR-155 (SKYC); Borneo, Sabah, Tawau, Gunong Rara 9.XI.1996, K. Eguchi leg., Eg96-BOR-323 (SKYC, THNHM); Borneo, Sarawak, Sg. Segerugok, Song, 22.IX.1993, A. Rahman leg. (SKYC); Borneo, Sarawak, Semengoh N.P., 18.IV.1993, Sk. Yamane leg. (SKYC, THNHM); Borneo, Sarawak, Miri, Lambir N.P., Head Quarter, 17.VIII.1995, H. Okido leg. (SKYC, THNHM); same loc., 8 ha plot, 30.VI.2004, Sk. Yamane leg., SR04-SKY-38 (SKYC, THNHM). **BRUNEI:** Tasek Merimbun, 17.II.1999, A. Tuah leg., Eg99-BOR-130 (SKYC, THNHM). **INDONESIA:** E. Kalimantan, Kutai N.P., Sangkimah, 8.IX.1993, Sk. Yamane leg. (SKYC, THNHM); Java, Ujung Kulou, Cibon, 15.III.1997, F. Ito leg., FI97–182 (SKYC, THNHM).

##### Distribution. 

Borneo (Sabah, Sarawak, Brunei, and E. Kalimantan) and Java (Fig. 12).

##### Bionomics.

All the members of the *Aenictus philippinensis* group are probably restricted to the Philippines except for *Aenictus punctatus* that is distributed on Borneo and Java. All of the materials of this species examined were collected from lowland rainforests. A colony from Sarawak was collected from rotten wood in September 1993. A colony from Lambir National Park (SR04-SKY-38) was collected at night.

##### Remarks.

This species is closely related to *Aenictus philippinensis*. See under *Aenictus philippinensis* for details.

#### Aenictus rabori

Chapman, 1963

http://species-id.net/wiki/Aenictus_rabori

Figs 11 12

Aenictus rabori Chapman 1963: 249, fig. 1.

##### Types.

**Figure 11.**
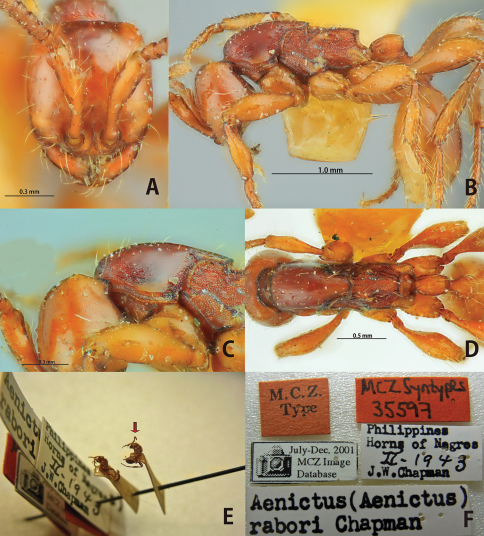
*Aenictus rabori* (lectotype). **A** Head in full-face view **B** habitus in profile **C** occipital corner of head **D** dorsal view of body **E** lectotype and paralectotype designated in the present paper (arrow indicating the lectotype) **F** labels of lectotype.

Nine syntype workers (two on each of three pins, three on another) from Philippines, Negros, Horns of Negros, 1,080 m (MCZC, examined). One worker among them (top on a pin) is selected as lectotype (Fig. 11E).

##### Measurements.

Lectotype: TL 4.50 mm; HL 0.85 mm; HW 0.83 mm; SL 0.65 mm; ML 1.48 mm; PL 0.35 mm; CI 97; SI 79.

Paralectotype (n = 8): TL 4.35–4.45 mm; HL 0.83–0.88 mm; HW 0.78–0.83 mm; SL 0.63–0.65 mm; ML 1.48–1.50 mm; PL 0.35–0.38 mm; CI 94–97; SI 76–79.

##### Description of worker

(lectotype and paralectotypes)**.** Head in full-face view slightly longer than broad, with sides convex and posterior margin nearly straight, very weakly sinuate; occipital margin bearing a distinct carina; occipital corner of head with a protruding lobe (part of occipital carina). Antennal scape relatively short, reaching only 2/3 of head length; antennal segment II slightly longer than each of III-VI; terminal segment slightly shorter than VII+VIII+IX. Frontal carinae short fused at the level of antennal base to form a single carina and much extending beyond the level of the posterior margin of torulus, poorly developed in posterior half. Parafrontal ridge short, extending less than 1/3 of head length, 0.25–0.27 mm long, seen in profile weakly developed in posterior half. Masticatory margin of mandible with a large apical tooth followed by a series of 4–5 denticles, which gradually reduce in size toward basal angle of mandible. Promesonotum in profile weakly convex dorsally and sloping gradually to metanotal groove; metanotal groove distinct and deep; upper portion of meso- and meta-pleuron impressed, much lower than promesonotum. Declivity of propodeum shallowly concave, encircled with a distinct rim that protrudes posteriad. Petiole subsessile, distinctly longer than high; subpetiolar process almost absent; postpetiole slightly shorter than petiole and almost as long as high, with its node rounded dorsally. Legs relatively short, seen from side with greatly swollen femora.

Head entirely smooth and shiny, except for hair pits, area on the side of head anterior to occipital corner with superficial reticulation; mandible extensively smooth and shiny except for hair pits; antennal scape superficially shagreened. Pronotum smooth and shiny, except for its anteriormost portion reticulate, narrow lateral margins distinctly reticulate, reaching back to posterior margin; mesonotum smooth and shiny; mesopleuron, metapleuron and propodeum densely punctuate/reticulate mixed with some rugae, mat except antero-ventral parts of meso- and metapleuron slightly shiny. Petiole microreticulate with dorsum more weakly sculptured; dorsum of postpetiole extensively smooth and shiny but other parts more or less reticulate. Femora entirely superficially reticulate and shiny; tibiae superficially shagreened partly smooth and shiny.

Head and mesosoma dorsally with relatively sparse long standing hairs; longest pronotal hair 0.25–0.27 mm long. Head, antennae, legs, and gaster yellowish brown; mandible, mesosoma, petiole, and postpetiole reddish brown.

##### Distribution.

**Figure 12.**
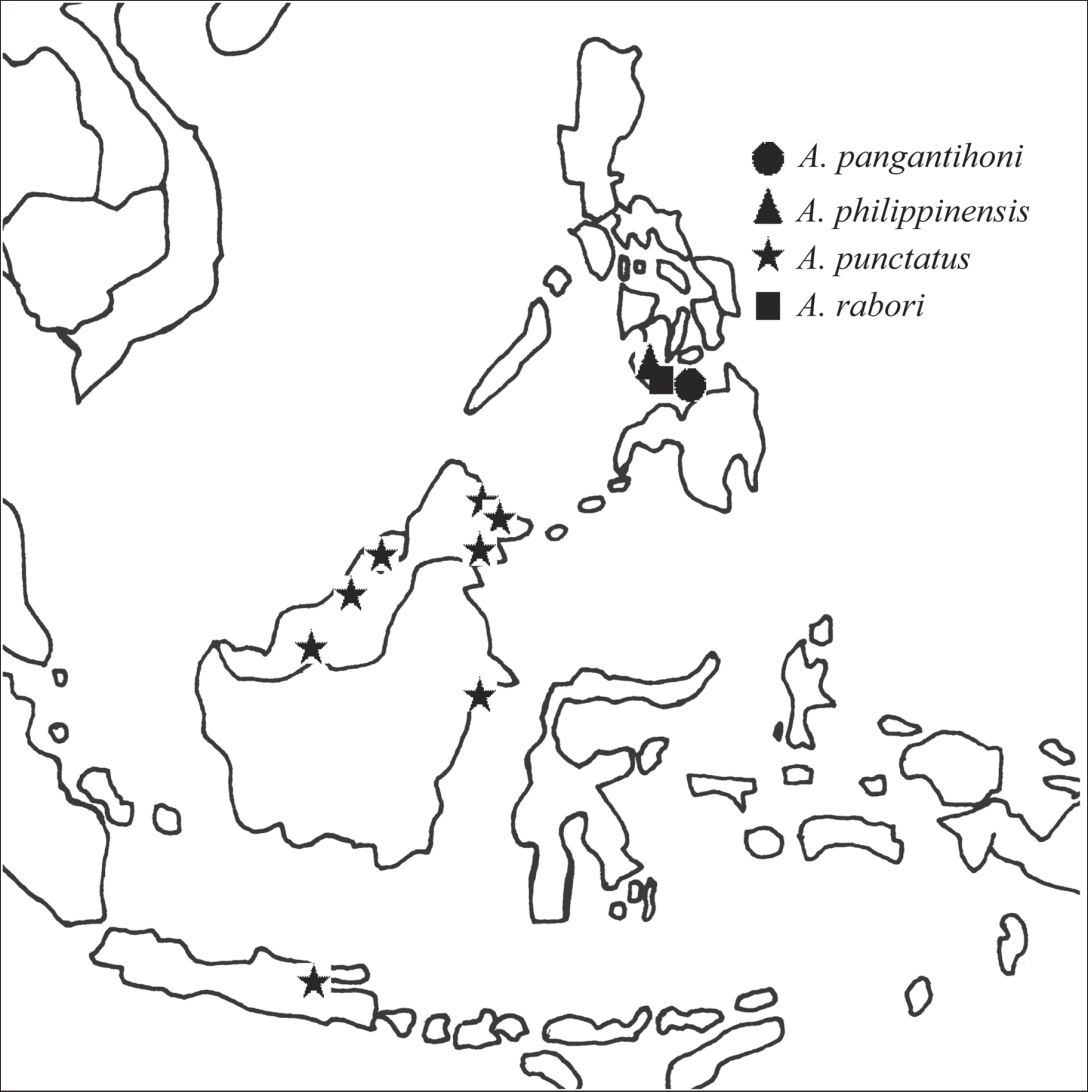
Distribution of the species of the *Aenictus philippinensis* group.

Philippines (Negros Island) (Fig. 12).

##### Bionomics.

So far *Aenictus rabori* is known only from the type locality**.** The colony observed by Chapman was foraging in a garden at 1,080 m in elevation (Chapman 1963).

##### Remarks.

This species is closely related to *Aenictus pangantihoni*. See under *Aenictus pangantihoni* for details.

## Supplementary Material

XML Treatment for Aenictus doydeei

XML Treatment for Aenictus duengkaei

XML Treatment for Aenictus javanus

XML Treatment for Aenictus longinodus

XML Treatment for Aenictus nishimurai

XML Treatment for Aenictus piercei

XML Treatment for Aenictus pangantihoni

XML Treatment for Aenictus philippinensis

XML Treatment for Aenictus punctatus

XML Treatment for Aenictus rabori
